# A study of size-dependent properties of MoS_2_ monolayer nanoflakes using density-functional theory

**DOI:** 10.1038/s41598-017-09305-y

**Published:** 2017-08-29

**Authors:** M. Javaid, Daniel W. Drumm, Salvy P. Russo, Andrew D. Greentree

**Affiliations:** 1 Chemical and Quantum Physics, School of Science, RMIT University, Melbourne, VIC 3001 Australia; 2 The Australian Research Council Centre of Excellence for Nanoscale BioPhotonics, School of Science, RMIT University, Melbourne, VIC 3001 Australia; 3 ARC Centre of Excellence in Exciton Science, School of Science, RMIT University, Melbourne, VIC 3001 Australia

## Abstract

Novel physical phenomena emerge in ultra-small sized nanomaterials. We study the limiting small-size-dependent properties of MoS_2_ monolayer rhombic nanoflakes using density-functional theory on structures of size up to Mo_35_S_70_ (1.74 nm). We investigate the structural and electronic properties as functions of the lateral size of the nanoflakes, finding zigzag is the most stable edge configuration, and that increasing size is accompanied by greater stability. We also investigate passivation of the structures to explore realistic settings, finding increased HOMO-LUMO gaps and energetic stability. Understanding the size-dependent properties will inform efforts to engineer electronic structures at the nano-scale.

## Introduction

Recently two-dimensional (2D) materials have drawn significant interest due to their unique structural, electronic, and optical properties^1–3^. The existence of 2D materials had been a highly debated issue until the successful exfoliation of graphene from graphite, the first experimentally stable 2D material^4^. After this revolutionary discovery, many other 2D materials such as silicene, hexagonal boron nitride, and transition-metal dichalcogenides (TMDCs) have also been exfoliated^5^. These 2D materials are now a widely growing field with a diverse range of applications in nano-electronics^3^.

Transition-metal dichalcogenides belong to a family of layered materials where each layer is connected through weak Van der Waals forces. They have a general formula of MX_2_, where M is a transition metal (M = Mo, W, Zr, Hf, *etc*.) and X is a chalcogen (X = S, Se, Te, *etc*.). Each layer is three atoms thick with the metal in the centre and the chalcogen atoms above and below the metal^6^. Nanoflakes of these materials are promising due to the properties emerging from their inter-layer or intra-layer bonding^7^. Property variations emerge by changing the number of layers or the lateral size within a layer. For example, bulk MoS_2_ has an indirect band gap of 1.2 eV but when it is thinned down to a single layer, its band gap switches to a direct band gap of 1.88 eV which makes it promising for electronic applications^8, 9^.

Molybdenum disulphide is a compound which belongs to the hexagonal *P*6_3_/*mmc* space group. In its layered structure, each S atom is covalently bonded to three Mo atoms and each Mo atom to six S atoms forming a trigonal prismatic coordination^10^. The symmetry group of monolayer MoS_2_ is ${D}_{3h}^{1}$ which contains the discrete symmetries: *C*_3_ trigonal rotation, *σ*_*h*_ reflection by the *xy* plane, *σ*_*v*_ reflection by the *yz* plane, and all of their products^11^.

There have been significant efforts to understand the size- and edge-dependent, structural and electronic properties of MoS_2_ monolayer nanoflakes. For example, quantum confinement effects in TMDC nanoflakes have been investigated by Miró *et al*., both experimentally and through density-functional theory (DFT)^7^. Wendumu *et al*. have presented the size-dependent optical properties of 1.6 to 10.4 nm MoS_2_ nanoflakes^12^ using the density-functional tight-binding (DFTB) method. An extensive DFT edge-dependence study on MoS_2_ monolayer nanoribbons has been reported by Pan *et al*.^13^. Ellis *et al*. have studied the band gap tranistion in multilayered MoS_2_ using DFT in gaussian09 with periodic boundary conditions^14^. Recently Nguyen *et al*. have experimentally studied the size-dependent properties of few-layer MoS_2_ nanosheets and nanodots^15^ but a complete study of the structural and electronic properties of very small single-layer MoS_2_ nanoflakes has not yet been presented.

**Figure 1:**
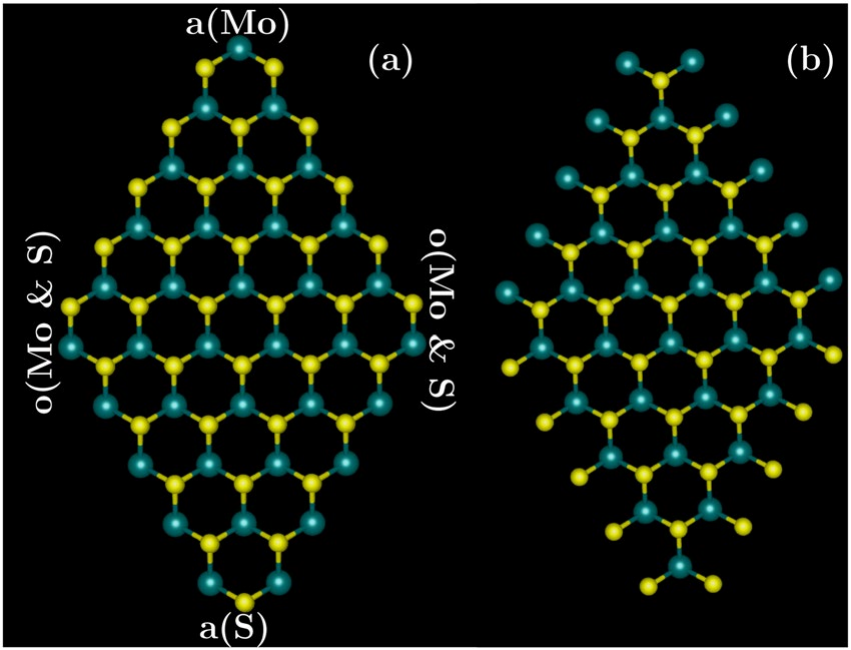
Nanoflakes of MoS_2_ monolayer having 105 atoms before geometry optimization: (**a**) zigzag edge configuration; (**b**) armchair edge configuration. Large, green atoms are Mo and small, yellow are S. Corner labels are defined as: a(Mo) = acute-Mo; a(S) = acute-S; o(Mo & S) = obtuse-Mo and S.

Here we report a DFT study of the 0 K size-dependent properties of 1H MoS_2_ monolayers of size smaller than 2 nm. We begin our discussion by studying the relative stability of the armchair and zigzag configurations shown in Fig. 1. We present the geometries of the relaxed structures for different nanoflake sizes to thoroughly understand the structural response as a function of lateral size. We report the electronic properties: binding energy, flake formation energy, HOMO-LUMO (highest-occupied molecular orbital to lowest-unoccupied molecular orbital) gap, charge densities; and the passivation of the flakes. We are particularly interested in exploring how the HOMO-LUMO gap changes with the nanoflake size, leading to applications in HOMO-LUMO gap engineering. This is especially important as the HOMO-LUMO gap is the first step in determining the tunable fluorescent properties of nanoflakes, and as MoS_2_ is known to be biocompatible^16^, nanoflakes of known size could be useful for biolabelling applications^17^.

This paper is organized as follows: first we discuss all the required methods and techniques. Then we study two different edge configurations for MoS_2_ monolayers and find the most stable one, following with a discussion of structural stability as a function of size, the electronic properties and the properties of the passivated structures.

## Methods

We investigated the structural and electronic properties of neutral MoS_2_ monolayer nanoflakes with stoichiometry Mo_*n*_ S_2*n*_ using DFT in gaussian09^18^. In experiments, usually triangular shaped islands of MoS_2_ have been reported but it has been theoretically speculated that MoS_2_ islands can exist in various shapes, such as trigonal, hexagonal, truncated hexagonal and rhombohedral^19–22^. We used rhombic flakes to maintain the neutrality and Mo_*n*_ S_2*n*_ stoichiometry of the flakes. Also, we experienced convergence issues with triangular flakes.

To choose an appropriate functional for our modelling, we conducted an in-depth analysis of the functionals listed in Table 1. We picked a relaxed 72-atom flake as this was the largest size we could model with the B3LYP functional. We compared the relative atomic positions of each atom in the central zone of the 72-atom flake with the bulk structure (infinitely large and regular structure in all three dimensions)^23^. The displacement Δ*R*_*i*_ of each atom from the bulk position is defined as

1 $${\rm{\Delta }}{R}_{i}\equiv \sqrt{{({X}_{{{\rm{opt}}}_{i}}-{X}_{{\rm{bulk}}})}^{2}+{({Y}_{{{\rm{opt}}}_{i}}-{Y}_{{\rm{bulk}}})}^{2}+{({Z}_{{{\rm{opt}}}_{i}}-{Z}_{{\rm{bulk}}})}^{2}},$$

where *i* indexes the atoms in the central zone of the 72-atom flake. The mean value of Δ*R*_*i*_, *i*.*e*., Δ*R* for each functional is given in Table 1. All functionals except B3LYP^24–26^ result in less than 5% variation from the bulk atomic positions. This indicates that the three functionals, BHandHLYP^27^, PBE1PBE^28^, and M052X^29^ predict similar structures at similar levels of accuracy.

**Table 1:** Mean displacement, Δ*R*, of atoms in the central zone of an optimized 72-atom flake from the bulk experimental positions of MoS_2_ using several functionals in gaussian09. All functionals except B3LYP predict mean displacements less than 5% from the bulk values.

Functionals	Δ*R*(Å)
PBE1PBE	0.0256
B3LYP	0.0565
BHandHLYP	0.0400
M052X	0.0330

**Figure 2:**
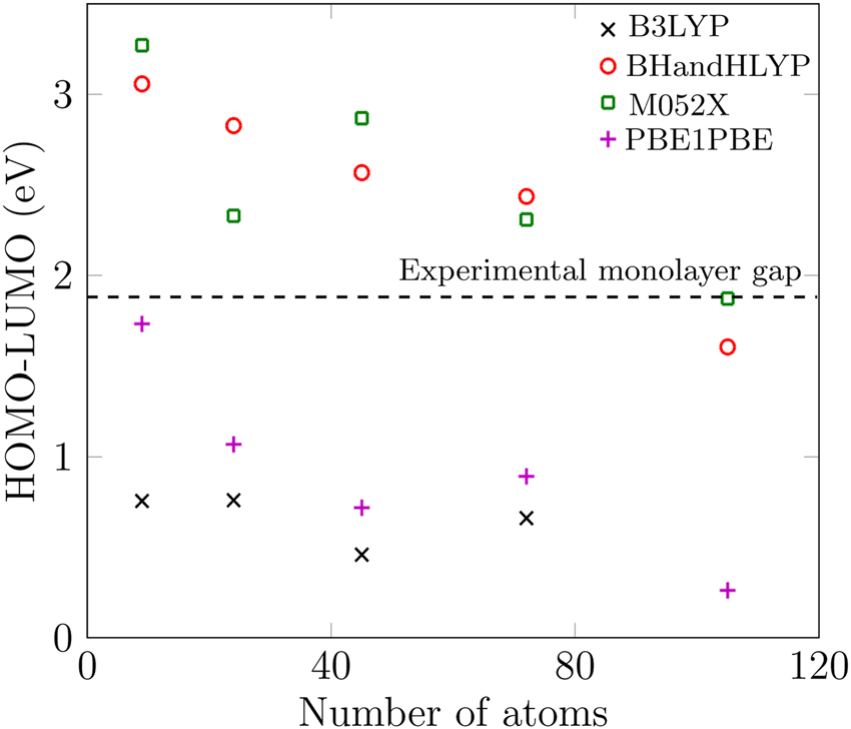
Size-dependent analysis of the HOMO-LUMO gap in MoS_2_ monolayer nanoflakes using four different functionals. The black-dashed line is the known experimental gap in a large sheet of the MoS_2_ monolayer^9^.

We also calculated the HOMO-LUMO gap as function of flake size for all these functionals as shown in Fig. 2. We expect the HOMO-LUMO gap to decrease with increasing flake size, approaching the experimental monolayer MoS_2_ gap for larger flakes, as reported by Gan *et al*.^30^ through an analytical equation for MoS_2_ monolayer quantum dots of size from 2 nm to 10 nm. Although our flakes are smaller than 2 nm and we are modelling in DFT, nevertheless we expect a similar trend of approximately decreasing bandgap with increasing flake size. Due to the different methods involved, we only compare the trends, not the absolute values of the HOMO-LUMO gaps. B3LYP and PBE1PBE produce HOMO-LUMO gaps well below the known experimental gap for a large MoS_2_ monolayer (Fig. 2). Hence, we do not consider these two functionals further. For smaller flakes, BH and HLYP and M052X both produce HOMO-LUMO gaps well above the monolayer experimental value^9^ and we can expect the band gap with these functionals to converge close to the experimental monolayer band gap for larger flakes. Cramer and Truhlar report that M052X is not a recommended functional for transition metal chemistry^31^. Considering this, we therefore used the BHandHLYP functional for this article, although we have also performed all the calculations with M052X functional and did not find any major difference in the results. A table showing the HOMO-LUMO responses of the smallest MoS_2_ monolayer nanoflake for several functionals (in the Supplementary information) also provided us with guidance for the optimal choice of functional for our DFT modelling.

The hybrid DFT functional, BHandHLYP^27^, includes a mixture of Hartree-Fock exchange with the DFT exchange-correlation via the relation

2 $${\rm{B}}{\rm{H}}{\rm{a}}{\rm{n}}{\rm{d}}{\rm{H}}{\rm{L}}{\rm{Y}}{\rm{P}}:\,0.5{E}_{x}^{{\rm{H}}{\rm{F}}}+0.5{E}_{x}^{{\rm{L}}{\rm{S}}{\rm{D}}{\rm{A}}}+0.5{\rm{\Delta }}{E}_{x}^{{\rm{B}}{\rm{e}}{\rm{c}}{\rm{k}}{\rm{e}}88}+{E}_{c}^{{\rm{L}}{\rm{Y}}{\rm{P}}};$$

${E}_{x}^{{\rm{HF}}}$ is the Hartree-Fock exchange term, ${E}_{x}^{{\rm{LSDA}}}$ is the Slater local exchange term^32^, ${\rm{\Delta }}{E}_{x}^{{\rm{Becke}}88}$ is Becke’s 1988^24^ gradient correction to the local-spin density approximation (LSDA) for the exchange term, and ${E}_{c}^{{\rm{LYP}}}$ is the Lee-Yang-Parr correlation term^25^.

The basis set used was an effective-core potential basis set of double-zeta quality, the Los Alamos National Laboratory basis set also known as LANL2DZ^33^ and developed by Hay and Wadt^34–36^. These basis sets are widely used in the study of quantum chemistry, particularly for heavy elements^33^.

gaussian09 optimization criteria: calculations were converged to less than 4.5 × 10^−3^ Hartree/Bohr maximum force, 3 × 10^−4^ Hartree/Bohr RMS force, 1.8 × 10^−3^ Hartree maximum displacement, and 1.2 × 10^−3^ Hartree RMS displacement. All the flakes were converged to the default SCF (self-consistent field) limit of <10^−8^ RMS change in the density matrix except those specified in the next section. The charge multiplicity (net charge) was 0 and the spin multiplicity was 1 (singlet; spin neutral).

In the geometry optimization process, the geometry was modified until a stationary point on the potential surface was found. Analytic gradients were used and the optimization algorithm was the Berny algorithm using GEDIIS^37^. We calculated the electronic properties of the optimized structures. The charge densities were plotted in avogadro^38, 39^ from a compatible gaussian09 checkpoint file.

### Data availability

The datasets generated and/or analysed during the current study are available from the corresponding author on reasonable request.

### Size-dependent structural properties

The properties of MoS_2_ monolayers are often investigated under the assumption of an infinite slab and real effects arising due to the confinement and boundaries are ignored. A nanoflake is a monolayer with spatial dimensions less than 100 nm. The structural, electronic and optical properties of such nanoflakes will be strongly influenced by varying their lateral size.

We study the MoS_2_ monolayer nanoflakes for two commonly known edge structures, zigzag and armchair, to investigate the stable edge structure for smaller nanoflakes. Structures before geometry relaxation without any edge termination are shown in Fig. 1. Zigzag structures have double-coordinated, bridge-like S or Mo atoms on the edges [Fig. 1(a)], whilst armchair have single-coordinated, antenna-like S or Mo atoms [Fig. 1(b)]. We relaxed both of these types of structure, encountering convergence issues for the two larger structures (72 atoms and 105 atoms). We succeeded in getting convergence of <10^−7^ RMS change in the density matrix for the 72-atom structures in both zigzag and armchair edge configurations. For the 105-atom structure, we obtained convergence of <10^−5^ RMS change in the density matrix in zigzag edge configuration, but could not converge the 105-atom armchair edge configuration at all. This therefore, sets the maximum structure size in our calculations. In gaussian09, the energy change is not a criterion for convergence, however, the worst level of convergence for the largest structure, *i*.*e*., <10^−5^ RMS change in density matrix, typically corresponds to <10^−10^ Ha change in energy^18^. For the larger structures, we are more confident of the trends instead of the absolute values of energy.

**Figure 3:**
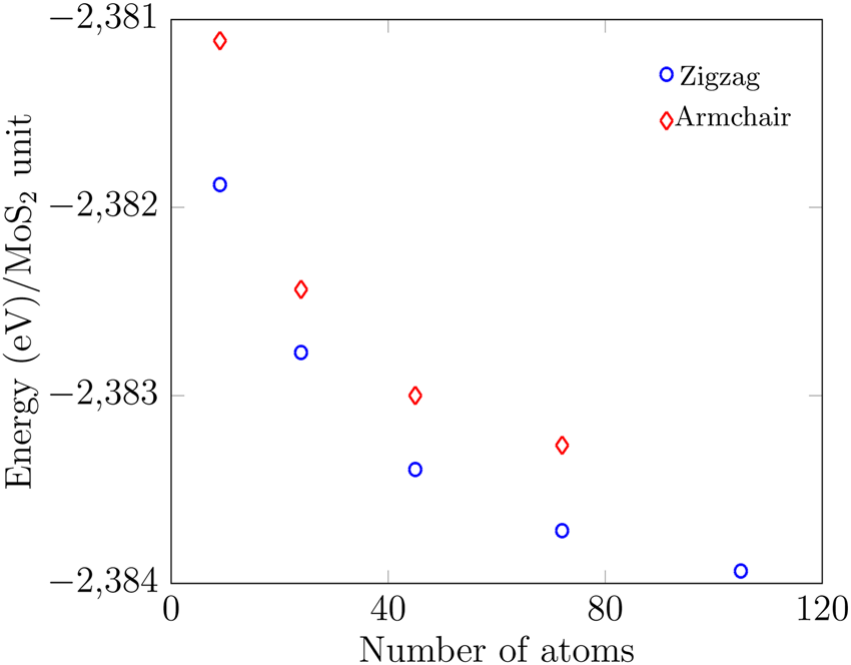
Ground-state energies as functions of size. Blue circles represent the zigzag edge configuration and red diamonds the armchair configuration.

The ground-state energies as functions of the size of the nanoflakes are shown in Fig. 3. Assuming that the edge width remains constant for any flake size, as the flakes get larger the ratio of number of edge atoms to core atoms decreases significantly because the number of core atoms increases more rapidly. (A quick circular approximation shows the core area ∝*L* ^2^, whilst treating the edge as an annulus gives area ∝*L*, where *L* is the radius of the core.) The structure becomes more stable as it becomes larger. Figure 3 shows that zigzag-edged structure is more stable than the armchair configuration for nanoflakes of size less than 2 nm. All further properties are discussed for the zigzag edge configuration only because it is the stablest.

**Figure 4:**
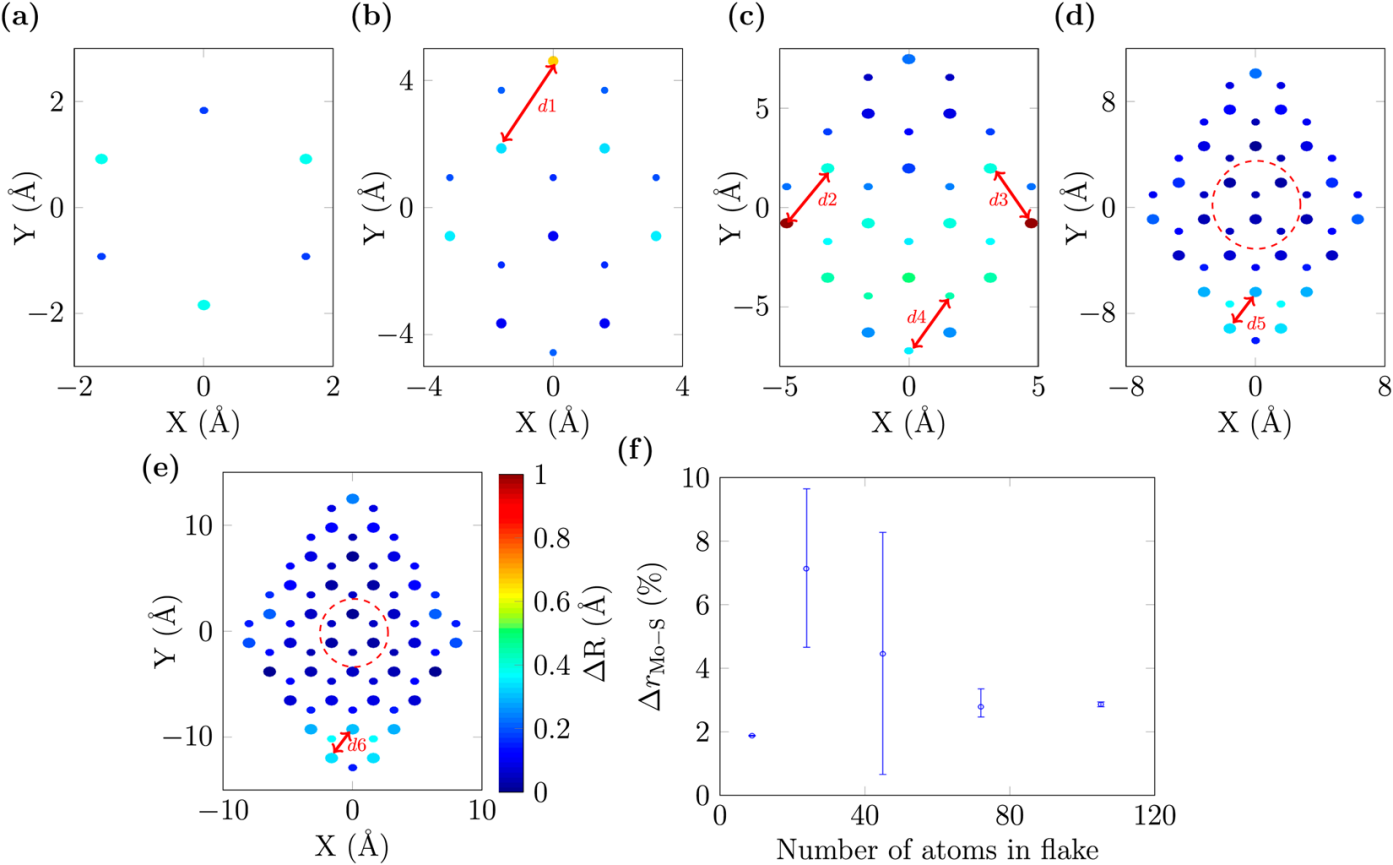
Relaxed structures of MoS_2_ monolayer nanoflakes comprised of: (**a**) 9 atoms, (**b**) 24 atoms, (**c**) 45 atoms, (**d**) 72 atoms, and (**e**) 105 atoms. The larger circles are Mo and the smaller are S. The colour of the atoms (Δ*R* given by Eq. (1)) represents variation of the atomic positions of relaxed structures from the bulk experimental positions^23^. S atoms are on top of each other along *z*-axis. The colour bar in (**e**) and the labels from Fig. 1(a) apply to all subfigures (**a**–**e**). The most distorted lengths in each flake are shown by the red-arrowed lines, *d*1*–d*6. (**f**) Percentage variation of the mean Mo–S bond length in the central zone of each flake from the bulk value^23^. Error bars are extended to the minimum and maximum Mo–S bond lengths in each central zone. The central zones are for (**a**) and (**c**) defined similar to that encircled red-dashed in (**e**), while for (**b**), it is similar to that encircled red-dashed in (**d**) as used previously in Table 1.

The relaxed structures of MoS_2_ monolayer nanoflakes are shown in Fig. 4. We compared the atomic positions in the relaxed structures with their unrelaxed positions in the bulk structure^23^. The colour of the atoms in this figure is proportional to the displacement of atoms from their bulk positions, Δ*R*_i_ as defined in Eq. (1), with *i* indexing all the atoms in the flakes.

The smaller nanoflakes are strongly distorted after relaxation compared to their unrelaxed structures except for the 9-atom flake. In the smallest structure having 9 atoms, all the Mo atoms are unsaturated symmetrically and all of them show the same distortion with a mean Mo–Mo length of 2.52 Å, while in the bulk structure this length is reported to be 3.15 Å^23^. Similarly all the S atoms show the identical distortion with S–S lengths of 3.43 Å. For the 24-atom structure, maximum distortion is observed at the acute-Mo [a(Mo)] corner. This maximum Mo–Mo length is shown by red-arrowed line *d*1 in Fig. 4(b), and is 2.66 Å. As we move to the next structure (45 atoms), this maximum distortion is shifted to the two obtuse-Mo & S [o(Mo & S)] corners. The unsaturated Mo atoms showing maximum distortion are displaced inwards [Fig. 4(c)]; for example, *d*2 and *d*3 are shortened to 2.50 Å while in the bulk structure, they are 3.15 Å. The maximum S–S length distortion in the same structure is *d*4 = 3.29 Å. As the structures get larger, we observe that the central zones show greatly reduced variation [Fig. 4(f)] after the optimization. For the two larger structures (with 72 and 105 atoms), the maximum distortion is shifted towards the acute-S [a(S)] corner ring [Fig. 4(d,e)]. Both of these structures show identical geometric behaviour and the maximum distortions are on the Mo–Mo lengths shown by red-arrowed lines *d*5 = *d*6 = 2.60 Å. These two structures show a well-established core whose mean structural parameters approach the bulk structure values^23^.

We have done an analysis of the Mo–S bond lengths in the central zones of our relaxed structures and compared them with the bulk Mo–S bond lengths of 2.41 ± 0.06 Å reported in^23^. Figure 4(f) shows the percentage variation of the mean Mo–S bond lengths in the central zone of each structure with the bulk Mo–S bond length, Δ*r*_Mo−S_ defined as:

3 $${\rm{\Delta }}{r}_{{\rm{Mo}}-{\rm{S}}}\equiv \frac{{r}_{{\rm{Mo}}-{\rm{S}}}^{{\rm{flake}}}-{r}_{{\rm{Mo}}-{\rm{S}}}^{{\rm{bulk}}}}{{r}_{{\rm{Mo}}-{\rm{S}}}^{{\rm{bulk}}}}\times \mathrm{100 \% }.$$

The error bars show the range of the minimum and maximum bond lengths in the central zone from the mean value. The smallest flake shows minimum mismatch from the bulk bond lengths. The flake with 24 atoms shows a mean mismatch of 5% from the bulk values. After that as the flake size increases, this percentage mismatch from the bulk values declines and then converges to a value of 2% [Fig. 4(f)] for the two larger structures.

### Size-dependent electronic properties

**Figure 5:**
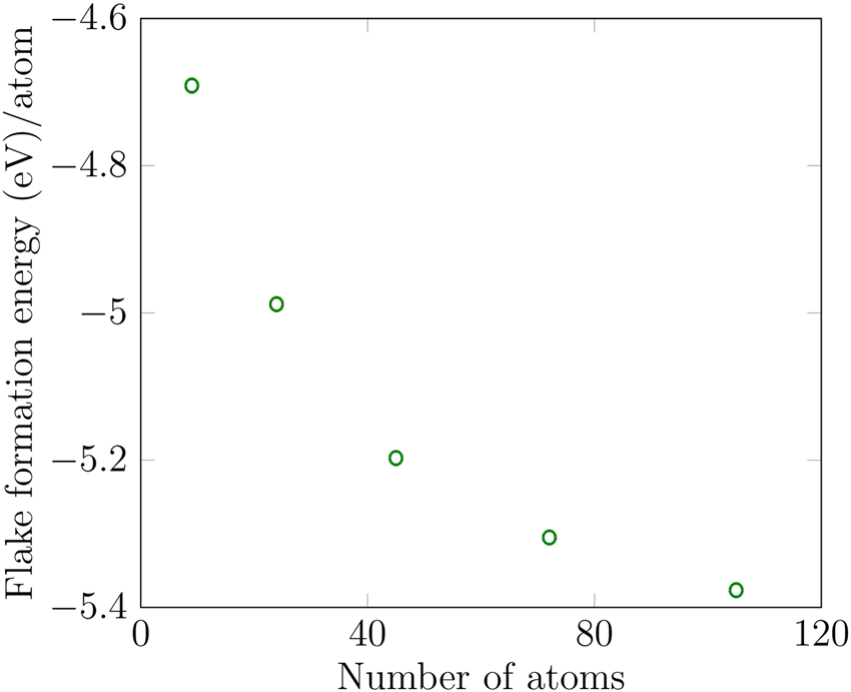
Flake formation energy as a function of nanoflake size. As the size increases, the formation energy decreases.

To indicate the stability and the tendency of flakes to grow, we calculated the size-dependent flake-formation energy (FFE) of MoS_2_ monolayer nanoflakes given by

4 $${\rm{FFE}}={E}_{{\rm{flake}}}({{\rm{Mo}}}_{n}{{\rm{S}}}_{2n})-nE({\rm{Mo}})-2nE({\rm{S}}),$$

where *n* is the number of Mo atoms and 2*n* the number of S atoms in the flake, *E*(Mo) is the energy of a single Mo atom, *E*(S) is the energy of a single S atom, and ${E}_{{\rm{flake}}}({{\rm{Mo}}}_{n}{{\rm{S}}}_{2n})$ is the energy of the flake having *n* Mo atoms and 2*n* S atoms. As defined, FFE < 0 indicates that the flake is more stable than its constituent atoms. Figure 5 shows that with the increase in nanoflake size the FFE decreases sharply, so more energy is released by adding atoms in the larger flakes indicating that the flakes tend to grow energetically. Conversely, more energy is required to break the larger flakes into their constituents.

**Figure 6:**
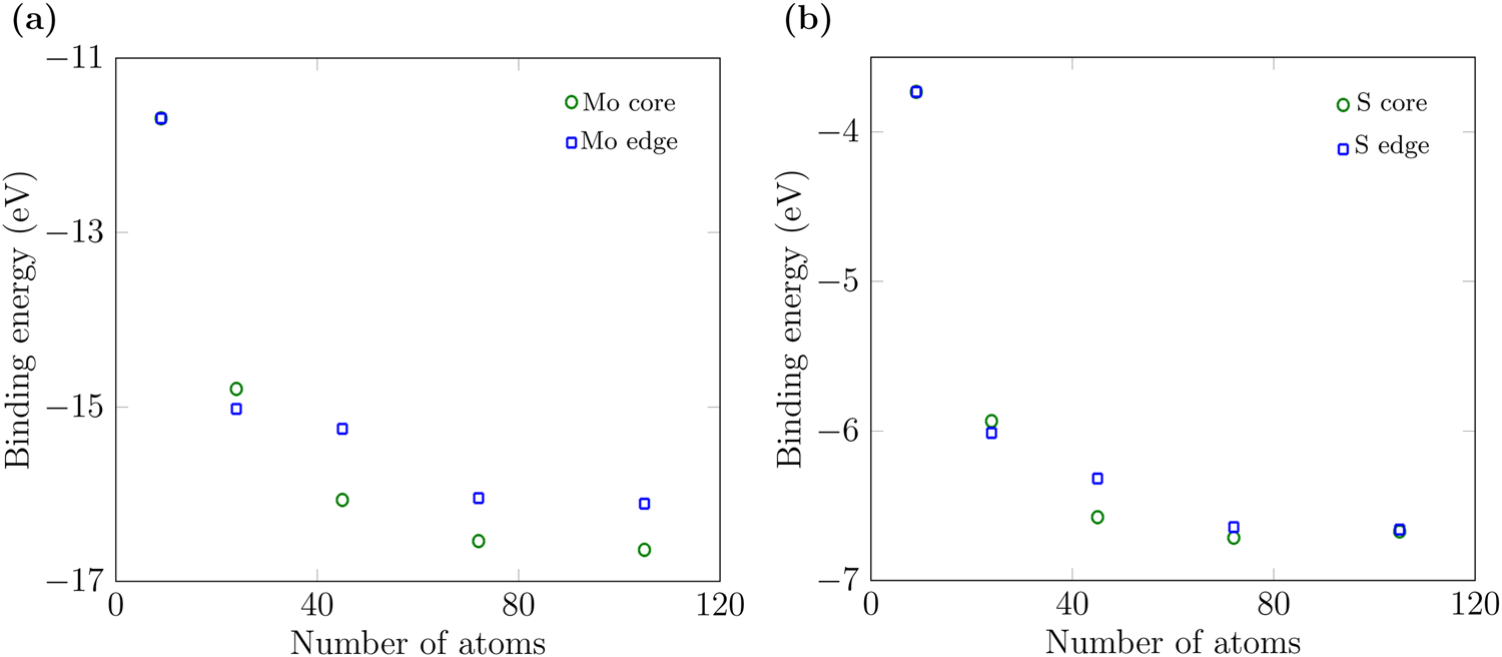
(**a**) Binding energies of Mo atoms as functions of number of atoms in the flakes. (**b**) Binding energies of S atoms as functions of number of atoms in the flakes.

We calculated the binding energies for all flake sizes and present them as a function of size in Fig. 6. We removed a Mo or S atom from as close as possible to the centre of the core or the edge as possible. The binding energy for the Mo atoms is given by

5 $${E}_{{B}_{{\rm{Mo}}}}=E({{\rm{Mo}}}_{n}{{\rm{S}}}_{2n})-E({{\rm{Mo}}}_{n-1}{{\rm{S}}}_{2n})-E({\rm{Mo}}\mathrm{)}.$$

Similarly, the binding energy for S atoms is given by

6 $${E}_{{B}_{{\rm{S}}}}=E({{\rm{Mo}}}_{n}{{\rm{S}}}_{2n})-E({{\rm{Mo}}}_{n}{{\rm{S}}}_{2n-1})-E({\rm{S}}\mathrm{)}.$$

Negative values of the binding energy indicate that energy is required to remove an atom from a nanoflake. The negative dependence with size means that the cost rises with flake size. For example, removing a Mo atom from the core of a 45-atom flake requires ~1.2 eV more energy than removing it from the core of a 24-atom flake. ${E}_{B}=-{E}_{{D}_{{\rm{form}}}}$, where ${E}_{{D}_{{\rm{form}}}}$ is the defect-formation energy so we can also calculate the energy required to create a Mo or S vacancy in the core or on the edge of the nanoflakes. From Fig. 6, significantly more energy is required to create a Mo vacancy as compared to a S vacancy. Also there is no major difference in the energy required to create a Mo vacancy in the core or in the edge in smaller flakes but as the size of the flakes increases, comparatively it becomes easier for defects to form on the edges. In case of S atoms, approximately the same energy is required to create a S vacancy in the core or in the edge as shown in Fig. 6(b).

**Figure 7:**
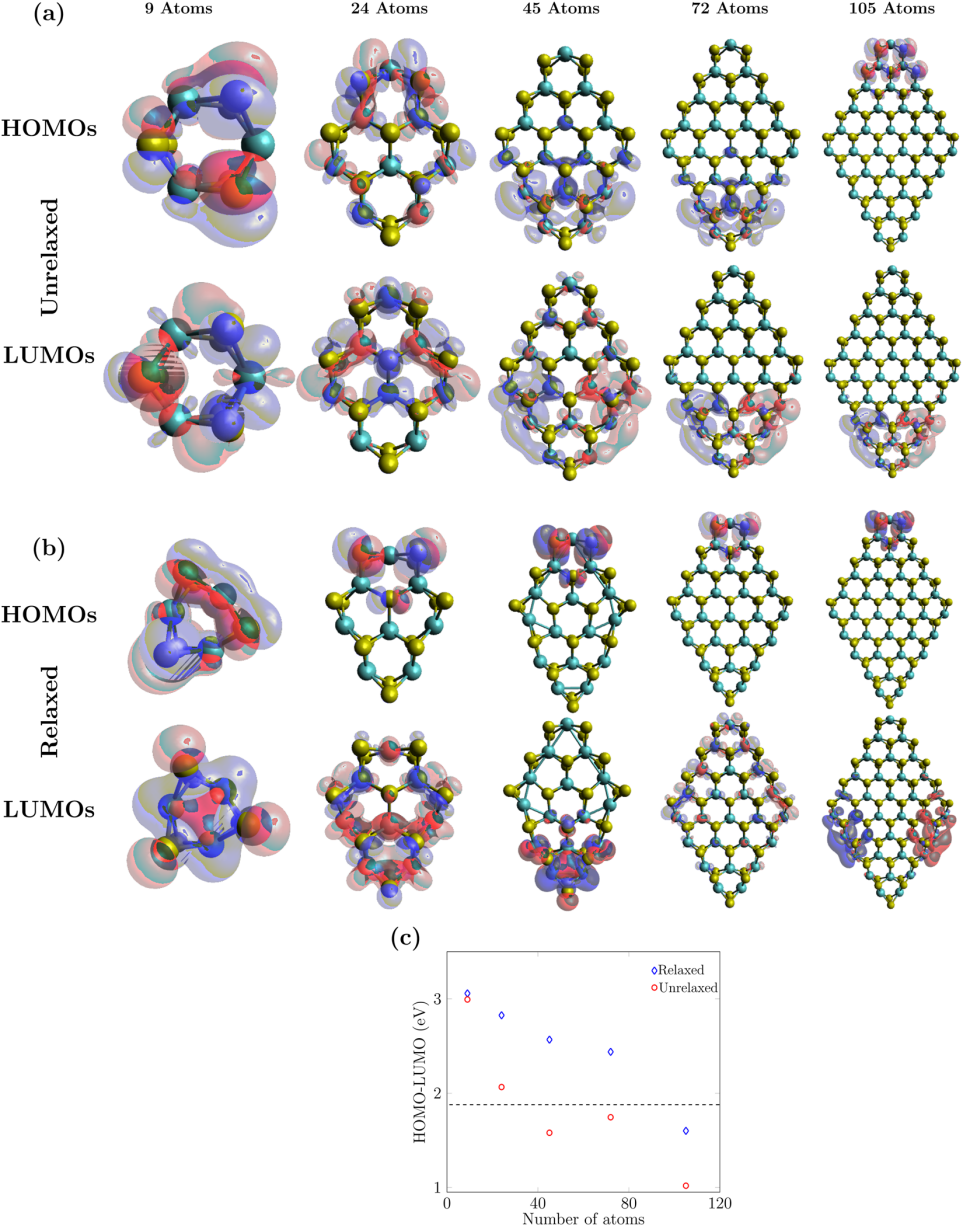
HOMO and LUMO charge densities of (**a**) unrelaxed, and (**b**) relaxed zigzag nanoflakes for various flake sizes at an isosurface value of 0.02 *e*/Bohr^3^. (**c**) HOMO-LUMO gaps as functions of size of the nanoflakes for both unrelaxed and relaxed structures. As the size of the nanoflakes increases, the gaps generally decrease. The black-dashed line indicates the known experimental band gap for a large sheet of MoS_2_ monolayer^9^.

To predict the electronic properties of ultra-small MoS_2_ monolayer nanoflakes, we calculated their HOMO-LUMO gaps and charge densities of their HOMO and the LUMO (Fig. 7). With an increase in flake size, the HOMO-LUMO gap decreases for both unrelaxed and relaxed structures which is in keeping with intuition around the increase in the HOMO-LUMO gap with decreasing particle size as discussed in the methods section. Mak *et al*.^9^ measured the band gap of 1.88 eV for a large MoS_2_ monolayer sheet as shown by the dashed line in Fig. 7. For larger flakes, we have not observed the band gap converging to this value. One possible cause could be dangling bonds in the nanoflakes. To address this, we study passivated structures in the next section.

To get deeper insight into the HOMO-LUMO behaviour as a function of nanoflake size, we calculated charge-density plots (Fig. 7) for structures before and after the geometry relaxation. We can see that the majority of the HOMO and the LUMO charge densities are lying on the corners and edges in all of these structures except the 9-atom nanoflake where they are scattered over the whole structure. No single, stand-out trend is observed across all the structures. In short, the charge density is highly sensitive to the structural size for these small sized nanoflakes.

### Hydrogen passivation of molybdenum-disulphide nanoflakes

Dangling bonds exist on the edges and corners of the nanoflakes. The smallest structure with 9 atoms has no fully coordinated atoms. The structure with 24 atoms possesses 5 under-coordinated Mo and 10 under-coordinated S atoms. Similarly, the structures with 45, 72, and 105 atoms possess 7 Mo and 14 S, 9 Mo and 18 S, and 11 Mo and 22 S under-coordinated atoms respectively.

It has been reported that the edge Mo atoms with unsaturated bonds may not be stable^20, 21^. Also in^40^, Topsoe *et al*. have reported the presence of S–H groups on the edges of MoS_2_ clusters experimentally. In ref. 41, Loh *et al*. have also passivated the S with H atoms in their triangular MoS_2_ quantum dot on hexagonal boron nitride substrate.

**Figure 8:**
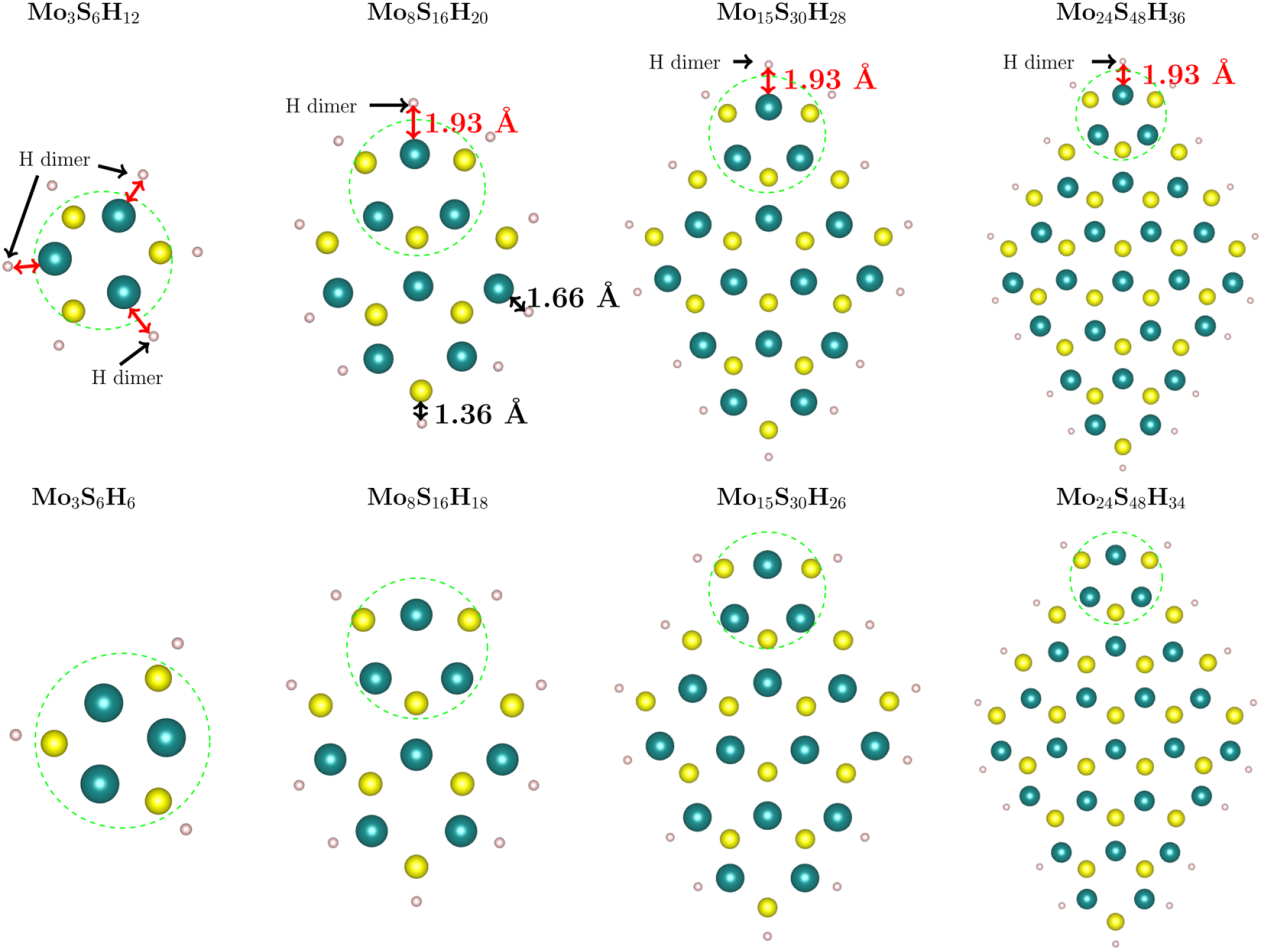
MoS_2_ monolayer nanoflakes passivated with H atoms and relaxed. Each Mo edge atom is passivated with two H atoms and each S edge atom is passivated with one H atom. The top row shows nanoflakes with H dimers not bonded to the flake (labelled). We removed these H dimers, relaxed the nanoflakes again and the relaxed structures are shown in the bottom row. The mean Mo–Mo, S–S lengths and Mo–S bond lengths in the green-encircled ring of each flake are reported in Table 2.

To understand the effects of dangling bonds on the properties of the structures, we passivated both Mo and S edges with H atoms. We passivated each edge Mo atom with two H atoms as we expect Mo atoms to be bonded with six atoms in this particular MoS_2_ stoichiometry. We also tested single H-termination of all edge Mo atoms and could not obtain converged, relaxed structures. We suspect this means that such structures are energetically unfavourable. We terminated each edge S atom with one H atom as all the central S atoms form three bonds with their neighbouring Mo atoms. We relaxed these passivated structures and observed that on the acute-Mo corner of all the nanoflakes, the H atoms are pushed away and they do not appear to bond to Mo atoms (Fig. 8). We investigated this non-bonding of corner Mo atoms with H atoms by checking their bond lengths. The average Mo–H bond length for all the edge Mo atoms is 1.665 ± 0.005 Å while on the corner it is 1.94 Å. The two H atoms on the Mo corner have an H–H bond length of 78 pm. We calculated the H–H bond length in a lone H dimer as 74 pm which is in good agreement with the known value^42^. The H–H bond length value, *i*.*e*., 78 pm on the acute-Mo corner in all passivated flakes is close enough to the known H–H value that we can believe that they are making a separate H_2_ molecule.

We removed the acute-Mo corner H atoms, relaxed the structures again and observed almost the same structural parameters on the corner as with the corner H atoms. We compared the mean Mo–Mo, S–S, and Mo–S lengths of the acute-Mo corner ring (encircled by green in Fig. 8) in Table 2 for the relaxed structures with and without the H dimer on the corner Mo atom. For all the structures, there is a minimal change in the bond lengths between 0–2%. All the S atoms bond well to one H atom each with an average S–H bond length of 1.365 ± 0.005 Å. We could not obtain a relaxed, converged 105-atom (we are not counting the number of H atoms to keep the number of atoms in each flake consistent with the previous discussion) passivated structure.

**Table 2:** A comparison of the mean lengths in the relaxed, passivated structures with and without H dimers on the Mo corner rings, encircled by green on all the structures in Fig. 8.

Nanoflake size	Mean Mo–Mo (Å)		Mean S–S (Å)		Mean Mo–S (Å)	
	with H dimer	without H dimer	with H dimer	without H dimer	with H dimer	without H dimer
9 atoms	2.41	2.40	3.65	3.74	2.60	2.60
24 atoms	2.73	2.69	3.48	3.52	2.53	2.54
45 atoms	2.72	2.69	3.51	3.55	2.53	2.53
72 atoms	2.73	2.70	3.52	3.54	2.53	2.53

There is a maximum mismatch of 2% in the S–S length in the 9 atom structure.

**Figure 9:**
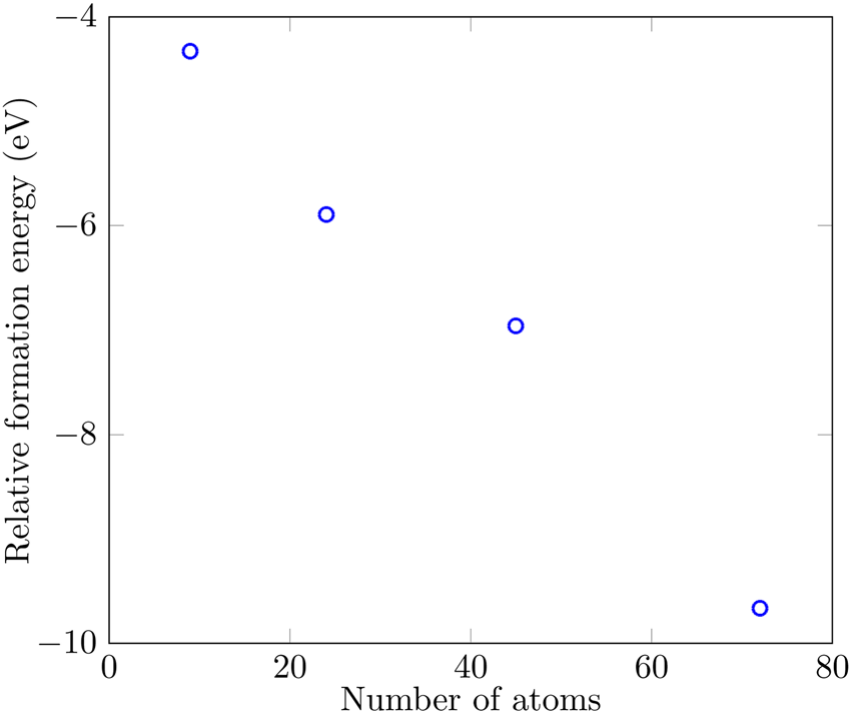
Energy difference between the passivated and unpassivated structures. The passivated structures are significantly more stable than the unpassivated ones in all cases.

To calculate the stability, we have compared the energies of the passivated structures with the corresponding unpassivated ones. We found that the passivated structures are significantly more stable than the unpassivated ones by 4.33, 5.9, 6.96, and 9.66 eV for 9, 24, 45, and 72 atoms respectively as shown in Fig. 9 where the relative formation energy (RFE) is:

7 $${\rm{RFE}}=E({{\rm{Mo}}}_{n}{{\rm{S}}}_{2n}{{\rm{H}}}_{m})-E({{\rm{Mo}}}_{n}{{\rm{S}}}_{2n})-\frac{m}{2}{{\rm{H}}}_{2},$$

where *m* is the number of H atoms in the passivated structures.

**Figure 10:**
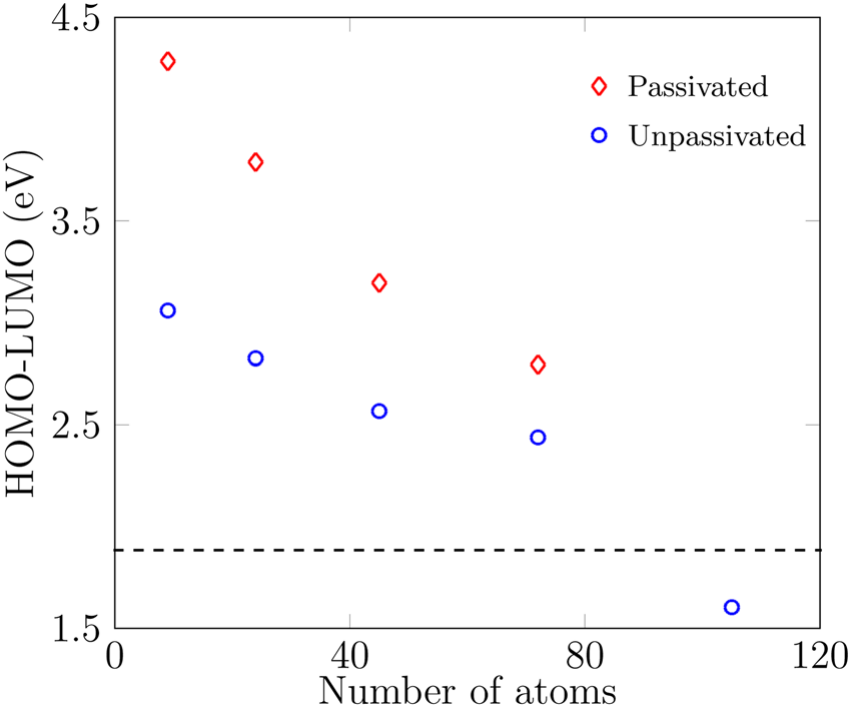
HOMO-LUMO gap of the unpassivated structures (blue circles) versus the passivated structures (red diamonds). Passivated structures have larger HOMO-LUMO gaps. The black-dashed line indicates the known experimental band gap of a large MoS_2_ monolayer sheet as reported by Mak *et al*.^9^.

Passivation of the dangling bonds modifies the electronic structure, charge densities, and hence the HOMO-LUMO gap. In Fig. 10, the HOMO-LUMO gap of the passivated structures is contrasted against the unpassivated ones. We find that the HOMO-LUMO gap widens with passivation. We suspect this is because of the removal of dangling bonds. This effect is significant in smaller nanoflakes but as the size increases, the ratio of edge to core atoms decreases. Hence, due to fewer edge states in the larger structures, the HOMO-LUMO gap difference (both relative and absolute) between the passivated and the unpassivated structures becomes smaller. The energy level diagram for the unpassivated and passivated flakes is shown in the Supplementary information.

**Figure 11:**
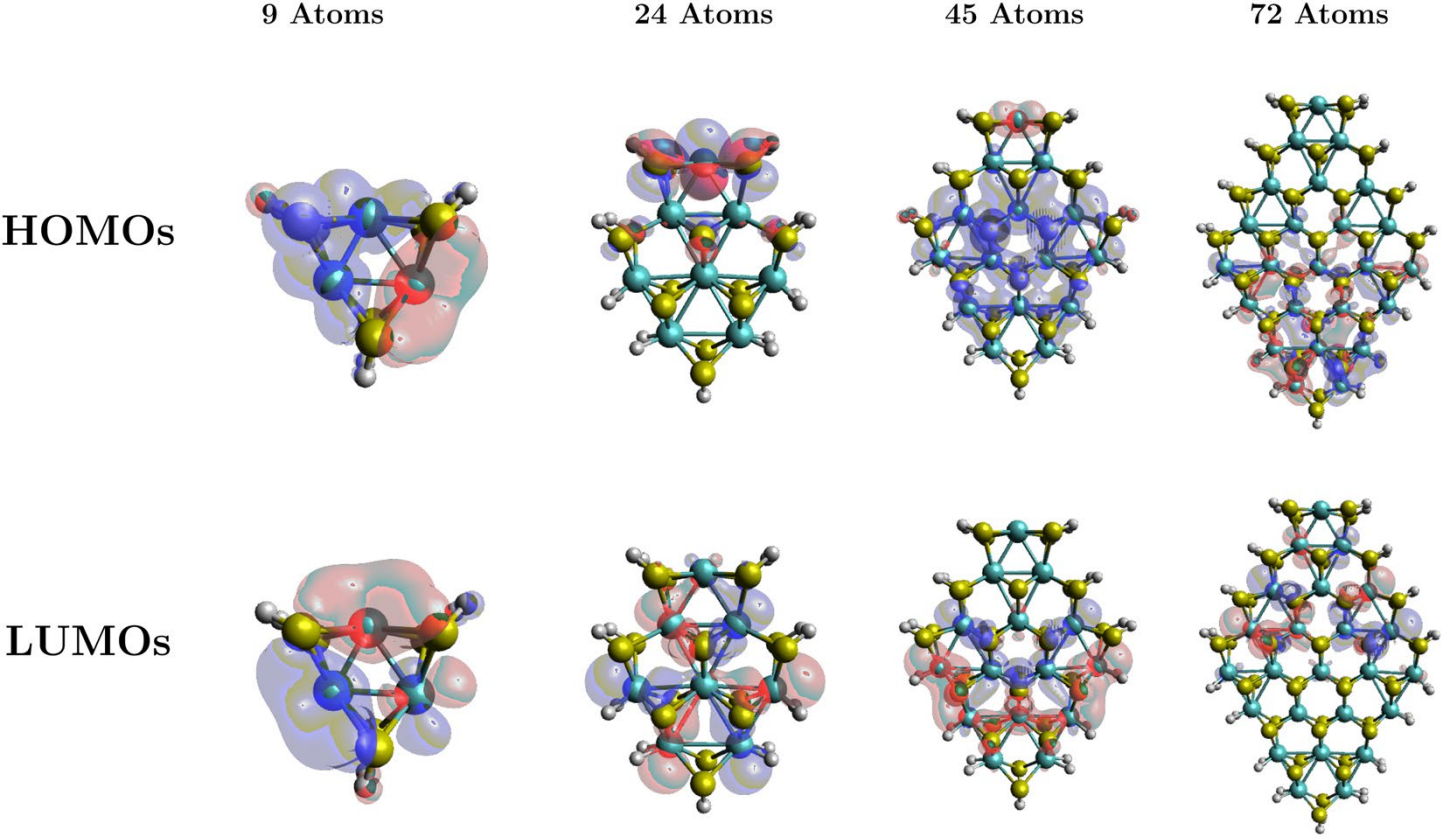
Charge densities in the HOMOs and LUMOs of the passivated structures for various sizes of nanoflakes for an isosurface value of 0.02 *e*/Bohr^3^. Charge densities are very sensitive to the size of the nanoflakes.

The charge densities of the passivated structures are shown in Fig. 11. These are much more distributed states in contrast to the charge density plots for unpassivated, relaxed structures [Fig. 7(b)]. Thus passivation makes HOMO/LUMO states in these small-sized flakes more like the expected infinite monolayer.

## Conclusions

In summary, we have investigated the size-dependent structural and electronic properties of MoS_2_ monolayer nanoflakes of sizes up to 2 nm using DFT. Our main focus has been to explore the small-sized nanoflakes. We provide more-detailed information for engineering small-sized nanoflakes by reporting the energetically favourable edge configuration and size of the nanoflakes. We predicted the trends in the energetics as functions of size. We passivated the structures to explore the effects of passivation on small-sized nanoflakes. We found the passivated structures to be more stable, with wider HOMO-LUMO gaps than unpassivated ones. We observe several strong size dependencies of various properties.

The size-dependence of the HOMO-LUMO gap of these small-sized nanoflakes holds promise for opto-electronic applications. However, due to the size-dependent energetics involved, one must take care in the manufacture/selection of these flakes. Due to limited computational resources, we were able to model only small-sized nanoflakes and can predict trends for larger flakes only by extending the fit functions. However, an extension of the current work to nanoflakes larger than 2 nm would be a good benchmark for the DFTB size-dependent HOMO-LUMO gaps reported by Wendumu *et al*.^12^.

## Electronic supplementary material

Supplementary Information
