# Supplementary material for: A study of size-dependent properties of MoS2 monolayer nanoflakes using density-functional theory
Source: Sci Rep. 2017 Aug 29;7:9775. doi: 10.1038/s41598-017-09305-y (PMC5575009; doi:10.1038/s41598-017-09305-y)
Supplement: Supplementary file 1 — Supplementary Information [file 41598_2017_9305_MOESM1_ESM.pdf]

A study of size-dependent properties of MoS_2 monolayer nanoflakes using density-functional theory

M. Javaid[1,2,*], Daniel W. Drumm[2], Salvy P. Russo[1,3], and Andrew D. Greentree[1,2]

[1]Chemical and Quantum Physics, School of Science, RMIT University, Melbourne VIC 3001, Australia
[2]The Australian Research Council Centre of Excellence for Nanoscale BioPhotonics, School of Science, RMIT University, Melbourne, VIC 3001, Australia
[3]ARC Centre of Excellence in Exciton Science, School of Science, RMIT University, Melbourne, VIC 3001, Australia
[*]maria.javaid@rmit.edu.au

Here we present a brief overview of the analysis of different functionals on a small MoS_2 monolayer nanoflake having 9 atoms. We also show energy-level diagrams for the passivated and unpassivated structures of various sizes to study the size-dependence of MoS_2 monolayer nanoflakes and the effects of passivation on the electronic energy levels of the nanoflakes.

To choose the appropriate functional for modelling these small-sized nanoflakes, we made a comparison of the HOMO-LUMO gap using different functionals in GAUSSIAN09 as shown in Table 1. We faced an energy convergence issue when using the BP86[1,2] functional and did not use it for further modelling as we suspected that the convergence issues would be worse for larger flakes using this functional. For HSEH1PBE[3–8], B3LYP[1,9,10], PBE1PBE[11], B3PW91[1,12], PBEh1PBE[13], and M05[14], we obtained gaps smaller than the known experimental band gap in infinitely large sheet of MoS_2 monolayer. We expect the HOMO-LUMO gap to decrease with increasing flake size and then converge to the infinite monolayer MoS_2 band gap for larger flakes as discussed in the main paper. Thus for these functionals, we expect the results to get worse with any increase in flake size. The M052X[15] and BHandHLYP[16] functionals predicted reasonable gaps for this small nanoflake and we can conjecture that they might asymptote near the experimental value for larger flakes.

Table 1. An analysis of the HOMO-LUMO gap in GAUSSIAN09 for a 9-atom nanoflake under different functionals.

Functionals	HOMO-LUMO gap (eV)
B3LYP	0.75
BHandHLYP	3.06
HSEH1PBE	0.25
BP86	Convergence error
B3PW91	1.44
PBE1PBE	1.73
PBEh1PBE	1.70
M05	0.67
M052X	3.27

In Fig. 1, we have shown the several energy levels from HOMO-4 to LUMO+4 for both passivated and unpassivated nanoflakes. The HOMO is scaled to zero on the energy axes for all flakes. In both passivated and unpassivated flakes, the HOMO-LUMO gap shrinks with increasing size as discussed in the main paper. In the unpassivated structures, from 9 atoms to 72 atoms, the conduction band gets significantly denser with increasing size, while there is no significant change in the valence band's level spacing. For 105 atoms, the level spacing in the conduction band increases slightly again.

In the passivated structures, the valence bands get denser with increasing flake size while oscillating behaviour is

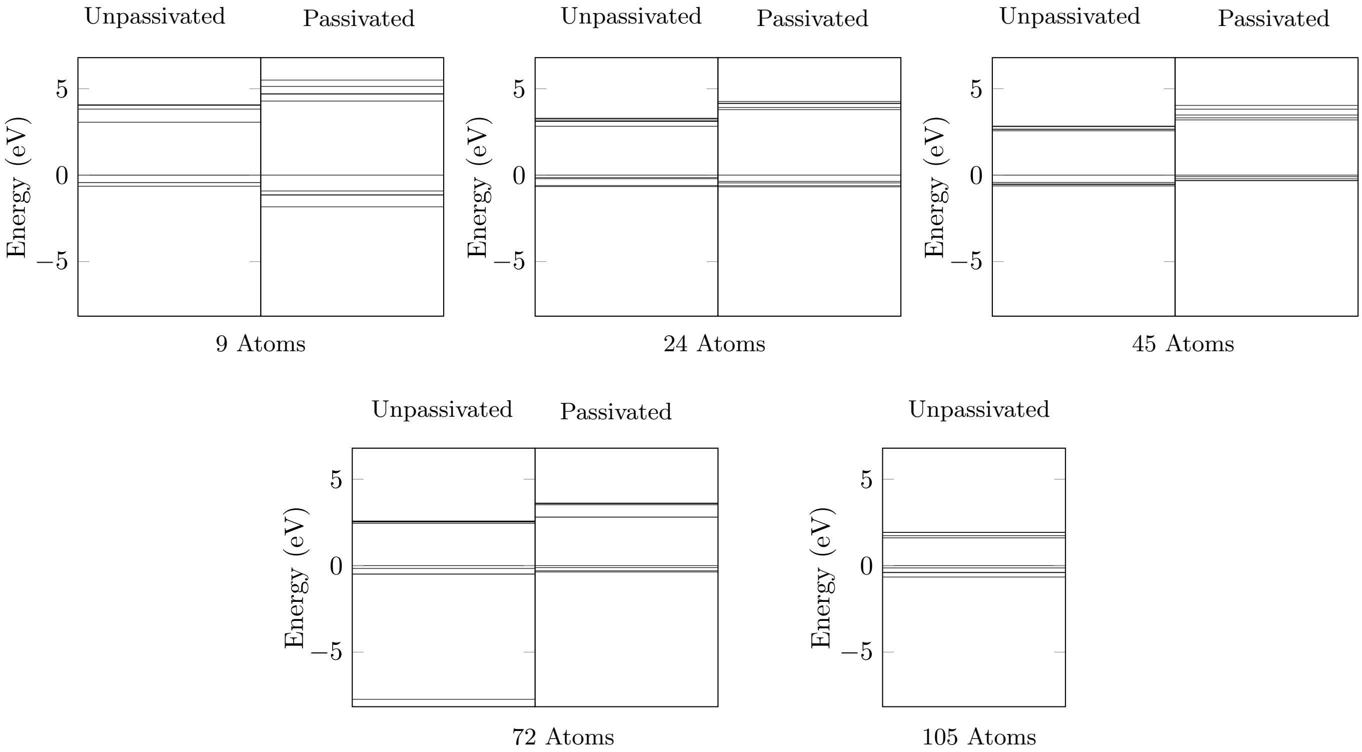

Figure 1. Energy levels from HOMO-4 to LUMO+4 in the unpassivated and passivated nanoflakes of various sizes. The HOMOs are scaled to zero on the energy axes.

observed in the conduction bands, which first gets denser from 9 atoms to 24 atoms, then slightly splits again for 45 atoms and then becomes denser again for 72 atoms. For all these structures, the HOMO-LUMO gap gets wider after passivation which is consistent with the idea that dangling bonds widen the band gap discussed in the main paper.

In summary, we obtained a reasonable subset of functionals to use for further modelling. We also found that the energy levels are very sensitive to the nanoflake sizes and that the dangling bonds play an important role in the HOMO-LUMO gap.

References

1. Becke, A. D. Density-functional exchange-energy approximation with correct asymptotic behavior. *Phys. Rev. A* **38**, 3098–3100 (1988).
2. Perdew, J. P. Density-functional approximation for the correlation energy of the inhomogeneous electron gas. *Phys. Rev. B* **33**, 8822–8824 (1986).
3. Heyd, J. & Scuseria, G. E. Efficient hybrid density functional calculations in solids: Assessment of the Heyd–Scuseria–Ernzerhof screened coulomb hybrid functional. *J. Chem. Phys.* **121**, 1187–1192 (2004).
4. Krukau, A. V., Vydrov, O. A., Izmaylov, A. F. & Scuseria, G. E. Influence of the exchange screening parameter on the performance of screened hybrid functionals. *J. Chem. Phys.* **125**, 224106 (2006).
5. Izmaylov, A. F., Scuseria, G. E. & Frisch, M. J. Efficient evaluation of short-range Hartree-Fock exchange in large molecules and periodic systems. *J. Chem. Phys.* **125**, 104103 (2006).
6. Henderson, T. M., Izmaylov, A. F., Scalmani, G. & Scuseria, G. E. Can short-range hybrids describe long-range-dependent properties? *J. Chem. Phys.* **131**, 044108 (2009).
7. Heyd, J., Scuseria, G. E. & Ernzerhof, M. Erratum: "Hybrid functionals based on a screened Coulomb potential" [j. chem. phys. 118, 8207 (2003)]. *J. Chem. Phys.* **124**, 219906 (2006).
8. Heyd, J., Peralta, J. E., Scuseria, G. E. & Martin, R. L. Energy band gaps and lattice parameters evaluated with the Heyd-Scuseria-Ernzerhof screened hybrid functional. *J. Chem. Phys.* **123**, 174101 (2005).

9. Lee, C., Yang, W. & Parr, R. G. Development of the Colle-Salvetti correlation-energy formula into a functional of the electron density. *Phys. Rev. B* **37**, 785–789 (1988).

10. Stephens, P. J., Devlin, F. J., Chabalowski, C. F. & Frisch, M. J. Ab initio calculation of vibrational absorption and circular dichroism spectra using density functional force fields. *J. Phys. Chem.* **98**, 11623–11627 (1994).

11. Adamo, C. & Barone, V. Toward reliable density functional methods without adjustable parameters: The PBE0 model. *J. Chem. Phys.* **110**, 6158–6170 (1999).

12. Becke, A. D. Density-functional thermochemistry III. The role of exact exchange. *J. Chem. Phys.* **98**, 5648–5652 (1993).

13. Ernzerhof, M. & Perdew, J. P. Generalized gradient approximation to the angle- and system-averaged exchange hole. *J. Chem. Phys.* **109**, 3313–3320 (1998).

14. Zhao, Y., Schultz, N. E. & Truhlar, D. G. Exchange-correlation functional with broad accuracy for metallic and nonmetallic compounds, kinetics, and noncovalent interactions. *J. Chem. Phys.* **123**, 161103 (2005).

15. Zhao, Y., Schultz, N. E. & Truhlar, D. G. Design of density functionals by combining the method of constraint satisfaction with parametrization for thermochemistry, thermochemical kinetics, and noncovalent interactions. *J. Chem. Theory Comput.* **2**, 364–382 (2006).

16. Becke, A. D. A new mixing of Hartree-Fock and local density-functional theories. *J. Chem. Phys.* **98**, 1372–1377 (1993).
